# Epidemiological, molecular and clinical features of Enterovirus 109 infection in children and in adult stem cell transplant recipients

**DOI:** 10.1186/1743-422X-9-183

**Published:** 2012-09-04

**Authors:** Maurizia Debiaggi, Elisa Rita Ceresola, Michela Sampaolo, Emilio Paolo Alessandrino, Roberto Brerra, Aurora Piazza, Massimo Clementi, Filippo Canducci

**Affiliations:** 1 Department of Morphological and Clinical Sciences, Section of Microbiology, University of Pavia, Pavia, Italy; 2 Laboratory of Microbiology and Virology, San Raffaele Scientific Institute and Vita-Salute, San Raffaele University, Milan, Italy; 3 Division of Hematology, Fondazione Istituto di Ricovero e Cura a Carattere Scientifico, Policlinico San Matteo, Pavia, Italy

**Keywords:** Enterovirus 109, Rhinoviruses, Acute respiratory disease

## Abstract

**Background:**

A novel human enterovirus (HEV) type within the species HEV-C, named EV109, was discovered from cases of respiratory illness in Nicaragua in September 2010. The aim of this study, was to retrospectively examine the presence and the role of EV109 in respiratory samples from two patients populations; infants below the age of 2 years, hospitalized for acute respiratory diseases (ARDs) and adult hematopoietic stem cell transplantation recipients.

**Results:**

A total of 1149 nasopharingeal aspirates were collected and tested for the presence of EV109 by reverse transcription-PCR (RT-PCR). In positive samples, the presence of the most common respiratory viruses was also assayed and clinical symptoms were evaluated. Samples from 2 of the 974 infants tested positive for EV109 RNA (0.2%) and belonged to patients with lower ARDs; co-infection with other viral pathogens under study was observed in both cases. In transplant recipients, one out of the 175 samples analyzed, from a patients with upper respiratory simptoms tested positive for HEV 109 in the absence of co-infecting viruses. Sequence analysis of amplified EV109 genomic regions, showed only a few nucleotide differences when compared with the Nicaraguan strains.

**Conclusions:**

Overall these results indicate that HEV109 variants have circulated and differentiated in different lineages worldwide. Although more cases and larger studies are needed, HEV109 infection may be associated to ARDs both in infants and in hematopoietic stem cell transplantation recipients. If these preliminary observations will be confirmed, improved molecular methods with a wider panel of potential pathogens will be useful for monitoring these categories of patients.

## Background

Human enteroviruses (HEV) belonging to the Picornaviridae family are ubiquitous viruses responsible for a wide range of clinical syndromes ranging from mild respiratory symptoms to serious conditions, including aseptic meningitis, encephalitis and acute flaccid paralysis. Some serotypes support respiratory infections, and it has been confirmed that they are responsible for bronchopneumonias and occasional fatal cases [1-4]. As for many RNA viruses, the genetic evolution of HEV occurs over time by means of point mutations and recombination phenomena allowing the virus to adapt to environment modifications and host selective pressure [2,5,6]. This genetic evolution is linked directly to virulence characteristics, tissue tropism and hence to the pathogenic potential of these viruses [5,7]. More than 100 different serotypes of non polio enteroviruses have been recognized and recently two new enterovirus, the HEV104 and the HEV109, classified within HEV species C, were reported in association with respiratory diseases [8].

HEV104 was identified in 2009 in Switzerland and later in other Countries often in association with respiratory signs [3,9]. HEV109 was molecularly identified in 2010 in the nose and throat swab samples collected between June 2007 and June 2008 from children enrolled in a cohort study of influenza-like illness in Nicaragua, in some cases also associated with enteric symptoms [10].

After the identification of these novel HEVs, another species C HEV distantly related to EV109 was retrieved from a rectal swab of a deceased patient during an outbreak of flaccid paralysis in Congo; in this sample poliovirus and other neurologic, enteric and respiratory viral pathogens were not detected [8].

The global distribution of HEV109 is currently unknown and no studies have been performed yet to evaluate the epidemiological features of this infection. Furthermore, extensive screening in different patients populations with different clinical syndromes is needed to establish the full spectrum of HEV109 disease association and evaluate its pathogenetic potential.

In this study we looked for the presence of HEV109 in respiratory samples from two patients populations; i) infants less than 2 years old, hospitalized for acute respiratory tract diseases (ARDs) and ii) adult immunosuppressed hematopoietic stem cell transplantation recipients where sequential nasopharyngeal aspirates were evaluated.

## Results

Respiratory samples obtained from 974 infants (age range 1 to 24 months; mean age 4 months) hospitalised from October 2004 to November 2010, were evaluated for the presence of HEV109. Diagnosis at admission was lower acute respiratory disease (L-ARD) in 759 of the 974 patients (78%) [bronchiolitis in 468 (48%), bronchitis in 156 (16%) and bronchopneumonia in 135 (14%)] and upper respiratory tract disease (U-RTD; including rhinitis, laryngitis and upper respiratory tract phlogosis) in the remaining 215 patients (22%). Samples from 2 of the 974 infants tested positive for HEV109 RNA (0.2%) and belonged to patients diagnosed with L-ARD. The two HEV109 positive children were 3 and 6 months old and samples were collected in November 2005 and January 2006. Clinical features of HEV109 positive infants are reported in Table 1. Co-infections with other viral pathogens were observed in both cases. In particular, in one case HRV was also identified and in the other HEV109 positive case a triple infection was observed with RSV and HRV. All samples taken from October 2004 to September 2006 had also been evaluated for the presence of RSV, hMPV, hCoV, hBoV and WU and KI Polyomaviruses genomes, in previous studies [11-13]. Of the 322 samples from this period, 90 (28%) were positive for RSV RNA, 48 (14.3%) for hMPV RNA, 28 for hCoV RNA (8.7%) [11 for OC43 (3.3%), 9 for NL63 (2.8%), 6 for HKU1 (1.9) and 1 for 229E (0.3%)] and 7 (2.2%) for hBoV DNA. Considering the 322 evaluations of this epidemic period, the prevalence of HEV109 resulted 0.6% and the virus circulated during the RSV epidemic peak contemporarily with hMPV and hCoV.

**Table 1:** Clinical features of EV109 positive infants and transplant patient

**Source**	**Age (months) sex**	**Time of sample colection**	**Symptoms**^**a**^**or signs**	**Clinical diagnosis**	**Comorbidity**	**Time in hospital (days)**	**Coinfecting viruses**
infant	3/M	November 01, 2005	fever	bronchiolitis	/	3	hRV
infant	6/M	January 19, 2006	hypoxia, fever	asthmatic bronchitis	/	6	RSV, hRV
Transplanted adult		February 2006, 180 days after transplantation	pharingodinia	pharingodinia			/

In the transplant recipient group, only one out of the 175 samples evaluated, tested positive for HEV109 infection. In particular, the presence of HEV109 was documented in a sample collected 180 days after transplantation in February 2006. Sixteen out of the 54 patients suffered of an episode of upper respiratory symptoms during the study (rhinorrhea, pharingodinia, tussis). From these patients, 58 samples were collected and 18 out of the 58 samples were collected during respiratory symptoms. The HEV109 positive patient was referring upper respiratory symptoms including pharingodinia lasting several days, but no gastrointestinal symptoms were recorded. Attempts to identify other co-infecting viruses including the HRV-C strain in the HEV109 positive sample gave negative results.

Notably, all three amplification protocols, including the HRV amplification protocol (that uses degenerated primers to amplify all rhinovirus and theoretically also several other Picornaviruses as suggested by sequence alignment and unpublished observations), used in the present study were able to detect HEV109 infection. Distinct agarose gel electrophoresis patterns and sequencing of individually gel extracted amplicons, allowed to exclude (in the transplant recipient sample) or confirm (in pediatric samples) co-infection of HEV109 with HRV (Table 1).

Unfortunately the low number of HEV109 positive samples could not allowed a reliable statistical analysis of the severity of the infection or co-infection in comparison to other respiratory viruses in both populations as described in previous papers for other recently identified pathogens [11,12].

**Figure 1:**
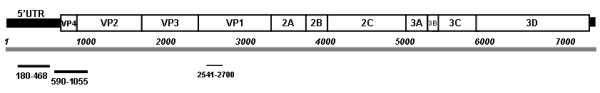
Mapping of the amplified regions on the reference sequence of Human enterovirus 109 (GenBank accession number: NC_014336).

**Figure 2:**
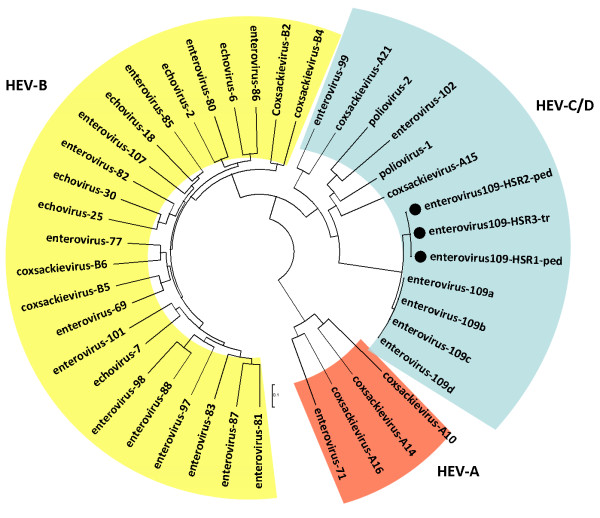
** Typing of HEV109 strains identified in pediatric patients and hematopoietic stem cell transplant recipients (indicated by black dots).** Phylogenetic relationships were estimated using neighbor-joining method with the Tajima-Nei model as described in the methods. Reference sequences were used to generate the alignment (http://www.picornastudygroup.com). Pediatric or transplant HEV109 strains are indicated with -ped or with -tr suffix respectively.

Analysis of 950 bases along the UTR, the VP4 and VP2 sequences (Figure 1) (GenBank accession numbers JX006784-9) indicated that, when compared, while the three identified HEV109 strains resulted almost identical (less than 1% nucleotide difference), HEV109 Nicaraguan strains resulted 6.5% and 4% different in nucleotide and amino acid sequences respectively when compared with our strains (Figure 2).

## Discussion

In February 2006 the novel HEV109 was identified in a symptomatic immunocompromised adult from a respiratory sample collected in Italy. This is the first report of this virus in a transplanted patient with respiratory symptoms. The retrospective evaluation carried out in a pediatric department in the same geographic area throughout a period of six years proves that in infants below two years old and hospitalised for ARDs, the circulation of EV109 was low (0.2%) and concentrated in a the same period (November 2005-February 2006) suggesting that the newly identified enterovirus may cause sporadic epidemics in northern Italy. The sequence analysis performed on 950 bases along the UTR, the VP4 and VP2 regions showed only a few differences in the nucleotide sequence of our strains compared with the Nicaragua strains. The UTR amplified region crosses the putative area of recombination that was observed in HEV109 and the limited variability observed suggests that this recombination is very old and that this strain has circulated worldwide [10].

Some results may point towards a possible role of this virus as co-pathogen in ARDs in infants. In fact, in the paediatric cases of this study, even if with other co-infections, EV109 was only detected in samples from infants with L-ARD. Moreover, we observed a very low difference in amino acid sequences between isolates of this study and those described in Nicaragua in symptomatic patients. However we must underling that since 78% of our pediatric samples were from infants with lower tract disease, we cannot exclude a population bias in our observations and more cases of HEV109 infections and larger studies are needed to definitively clarify this point. In fact no HEV109 single infections were observed in pediatric patients and the limited number of HEV109 cases didn’t allows a statistically significant comparison of HEV109 co-infection severity compared to HEV109 negative co-infections as we described in previous papers for other pathogens [11,12].

The tedious pharingodinia suffered by the adult immunocompromised patient where HEV109 was isolated in the absence of other co-infecting respiratory viruses suggesting a direct pathogenetic role of this virus as speculated in initial observations [10]. Other pathogens, including HRV infections were common and as previously documented for hMPV [13,14], frequently asymptomatic in this group of patients, including the majority of rhinoviruses infections due to HRV-C strain (data not shown). Respiratory syndromes were indeed not common in transplant recipients and only 16 out of 54 transplant patients suffered sporadic episodes of upper respiratory symptoms. As recently suggested however, since symptomatic and also asymptomatic viral infections can trigger acute rejection and obliterative bronchiolitis in lung transplant recipients [15] the role of this new HEV needs to be clearly elucidated.

## Conclusions

These results, even if only partial genome sequences were analyzed and larger cohorts may be needed, suggest that HEV109 variants have circulated and differentiated in different lineages worldwide and can be associated to ARDs both in infants and in hematopoietic stem cell transplantation recipients. Although these initial observations cannot be conclusive on the pathogenic potential of this novel strain, an always wider panel of potential pathogens may be needed for a continuous monitoring of immunocompromised patients and for a better epidemiological characterization of respiratory infections in infants.

## Methods

The present study was approved by the Istituto di ricovero e Cura a Carattere Scientifico (IRCCS) Policlinico San Matteo Ethical Committee, and informed consent was obtained from parents or guardians in compliance with the Helsinki Declaration and for the consent to publish individual clinical data (Table 1).

*Pediatric samples.* A total of 974 archived nasopharyngeal aspirates (NPA) from children <24 months old and hospitalized for ARDs (mainly bronchiolitis, pneumonia, bronchitis, bronchospasm or wheezing) were examined in the present study. Samples were collected from October 2004 to November 2010, at the microbiology laboratory of the Azienda Ospedaliera in Melegnano. Samples collected during the 2004–2006 period were part of previous studies to characterize the infections of human respiratory syncytial virus (HRSV) and other respiratory viruses identified recently in infants with ARDs [11,12]. In all cases NPAs were collected as part of the standard diagnostic practice to assess the presence of HRSV. Clinical data including comorbidities or subsequent bacterial infections, the duration of hospitalization, the presence of hypoxia, fever >38°C or gastrointestinal symptoms at the time of diagnosis were also available for most infants.

*Transplant recipients’ samples.* A total of 175 archived NPAs were obtained from 50 allogeneic and 4 autologous haematopoietic stem cell transplant recipient patients recruited at the Division of Hematology, IRCCS Policlinico San Matteo (Pavia, Italy) regardless of respiratory symptoms, as described [13,14]. Respiratory samples were consecutively obtained after informed consent from October 1st, 2004 to April 2007. One to nine samples from each patient were collected every 30 days up to 180 days after transplantation. At each time point clinical data were recorded.

All samples were extracted by using Qiagen RNA mini kit (Qiagen, Germany), in accordance with the manufacturer’s protocol.

To investigate the prevalence of HEV109 in children and haematopoietic stem cell transplant recipient patients, specific primers targeting the UTR region were used (EV109 VP1 123 F, 5′-GGA GAC TGG AGC AAC TAG TAA AG-3′; EV109 VP1 363R, 5′-GGT GAA CAT TTC CAA TTT CCT ACG-3′). To better characterize the novel HEV 109 strains the VP4/VP2 region was also amplified using the following primers: P2-4-Rw GCA TCI GGY ARY TTC CAC CAC CAN CC; VP2-4-Fw GGG ACC AAC TAC TTT GGG TGT CCG TGT (Figure 2). The sensitivity of amplification protocols was determined by cloning each amplified target regions into pCR2.1 plasmid vector (TA Cloning Kit; Invitrogen), serially diluted from 10^6^ copies to 1 copy as previously described [11,13,16] Amplification protocols ensured the detection of 5 and 15 DNA copies/reaction of HEV109 target regions respectively (data not shown).

After agarose gel electrophoresis visualization, all the amplification products were sequenced bidirectionally to confirm amplification specificity. Molecular identification and typing of HEV109 positive samples, were performed with MEGA 3.1 software after ClastalW alignment and manual sequence editing with BioEdit. Phylogenetic relationships were estimated using MEGA V3.1, (neighbor-joining method by using Tajima-Nei model as estimated by using Modeltest; the α value used in MEGA was previously estimated directly from the data by using PAUP).

Since multiple infections are frequently detected in respiratory samples of patients with respiratory symptoms and to better clarify the pathogenetic role of the novel enterovirus in co-infections, all specimens positive for HEV109 were also assayed for the presence of other respiratory viruses, including parainfluenza viruses (PIV 1–3), influenza A and B viruses, human metapneumovirus (HMPV), human respiratory syncytial virus (HRSV), adenoviruses, human coronaviruses (hCoV), human bocavirus (hBoV) and for WU (WUPyV) and KI (KIPyV) polyomaviruses using a multiplex PCR strategy (Seeplex RV12 ACE Detection, Seegene, Rockville) and in house protocols [11-13]. Presence of human rhinoviruses (HRVs) in HEV109 positive samples from infants and in all samples from immunosuppressed patients were also investigated by molecular analysis and phylogenetic reconstruction. Samples were subjected to first round one step reverse transcription and amplification (Super-ScriptIII One Step RT-PCR with Platinum Taq (Invitrogen) after RNA extraction using Primers P1-1 (CAA GCA CTT CTG TYW CCC C) and P3-1 (ACG GAC ACC CAA AGT AG) and the following thermal profile: 20 min 50°C, 10 min 95°C, followed by 15 'touchdown' cycles (The steps were 94°C for 20 s, 68°C down to 52°C (2°C intervals) for 30 s), and 25 cycles at 94°C 30 s, 52°C 30 s and a final extention step at 68°C for 40 s.; 5 μl of first RT-PCR reaction underwent a semi-nested amplification using the Platinum TaqDNA Polimerase (Invitrogen) and the forward primer P1-1 and three reversed primers P2-1 (TTA GCC ACA TTC AGG GGC), P2-2 (TTA GCC ACA TTC AGG AGC C) and P2-3 (TTA GCC GCA TTC AGG GG) as described previously with minor modifications [17]. In all amplification protocols primers were used at a final concentration of 200 nM. Amplification specificity was confirmed by sequencing and phylogenetic internal control used to exclude contaminations [18,19].

Since this amplification protocol amplified also HEV109 homologous target sequence, in co-infected samples, amplifications products after gel electrophoresis (data not shown) were individually gel extracted (using Qiagen mini elute gel extraction kit) and sequenced to confirm amplification specificity. In all HRV samples when visualized in the gel only the nested amplification product of about 300 bp was observed. In the co-infected samples instead, after both amplification reaction, an additional 390 bp band was indeed always present that when sequenced independently corresponded to the HEV109 genome amplified with the outer primers. We cannot exclude that a deeper clonal analyses of each band would have demonstrated the presence of both viruses in both amplification products, however in the mono-infected transplant recipient patient the inner amplification product corresponded to the HEV only when sequenced.

## Competing interests

The authors declare that they have no competing interests.

## Authors' contributions

ERC, MS, RB, AP performed the experiments EPA collected samples, MC, MD and FC analyzed data, FC and MD wrote the draft. All authors read and approved the final manuscript.
